# Cardiolipin prolongs the lifetimes of respiratory proteins in *Drosophila* flight muscle

**DOI:** 10.1016/j.jbc.2023.105241

**Published:** 2023-09-09

**Authors:** Mindong Ren, Yang Xu, Colin K.L. Phoon, Hediye Erdjument-Bromage, Thomas A. Neubert, Michael Schlame

**Affiliations:** 1 Departments of Anesthesiology, Physiology, New York University Grossman School of Medicine, New York, New York, USA; 2 Departments of Cell Biology, Physiology, New York University Grossman School of Medicine, New York, New York, USA; 3 Departments of Pediatrics, Physiology, New York University Grossman School of Medicine, New York, New York, USA; 4 Departments of Neuroscience and Physiology, New York University Grossman School of Medicine, New York, New York, USA

**Keywords:** insect, isotopic tracer, lipid–protein interaction, membrane biogenesis, mitochondrial respiratory chain complex

## Abstract

Respiratory complexes and cardiolipins have exceptionally long lifetimes. The fact that they co-localize in mitochondrial cristae raises the question of whether their longevities have a common cause and whether the longevity of OXPHOS proteins is dependent on cardiolipin. To address these questions, we developed a method to measure side-by-side the half-lives of proteins and lipids in wild-type *Drosophila* and cardiolipin-deficient mutants. We fed adult flies with stable isotope-labeled precursors (^13^C_6_^15^N_2_-lysine or ^13^C_6_-glucose) and determined the relative abundance of heavy isotopomers in protein and lipid species by mass spectrometry. To minimize the confounding effects of tissue regeneration, we restricted our analysis to the thorax, the bulk of which consists of post-mitotic flight muscles. Analysis of 680 protein and 45 lipid species showed that the subunits of respiratory complexes I-V and the carriers for phosphate and ADP/ATP were among the longest-lived proteins (average half-life of 48 ± 16 days) while the molecular species of cardiolipin were the longest-lived lipids (average half-life of 27 ± 6 days). The remarkable longevity of these crista residents was not shared by all mitochondrial proteins, especially not by those residing in the matrix and the inner boundary membrane. Ablation of cardiolipin synthase, which causes replacement of cardiolipin by phosphatidylglycerol, and ablation of tafazzin, which causes partial replacement of cardiolipin by monolyso-cardiolipin, decreased the lifetimes of the respiratory complexes. Ablation of tafazzin also decreased the lifetimes of the remaining cardiolipin species. These data suggest that an important function of cardiolipin in mitochondria is to protect respiratory complexes from degradation.

While most cellular proteins are continuously degraded and resynthesized, a subset of long-lived proteins has emerged mostly in the nucleus and in mitochondria (1, 2, 3, 4, 5, 6, 7). In postmitotic tissues, such as the brain, muscle, and heart, such proteins may persist for months. The function of long-lived proteins is still being debated. However, many of them belong to large protein complexes that might be essential to cellular integrity (3, 4). In mitochondria, these are primarily the complexes of oxidative phosphorylation (OXPHOS), which reside in cristae, the densely packed invaginations of the inner membrane (6, 7, 8). The presence of long-lived proteins in mitochondria may seem surprising, given the dynamic nature of this organelle. It has been proposed though that long-lived protein complexes provide structural stability to continuously changing mitochondria, akin to weight-bearing walls in a building under reconstruction (3, 6). If longevity is indeed important, the question arises by what mechanisms long-lived proteins are preserved against the omnipresent forces of protein degradation.

In addition to OXPHOS proteins, the inner membrane of mitochondria contains a specific phospholipid called cardiolipin (CL). It has been demonstrated that CL is physically associated with OXPHOS proteins through non-covalent interactions and that its presence is critical to optimize OXPHOS activity (9, 10). Interestingly, the half-life of CL is substantially longer than the half-life of most other phospholipids (11, 12, 13). Thus, CL may be called a long-lived lipid in analogy to long-lived proteins.

The co-localization of long-lived proteins (OXPHOS complexes) and long-lived lipids (CL species) in mitochondrial cristae, raises the question of whether their lifetimes are similar and whether their longevities have a common cause. In this article, we measured the half-life times of proteins and lipids in *Drosophila melanogaster* with stable isotopes. *Drosophila* is an ideal organism for lifetime studies because flies do not change their body mass during adulthood, which precludes the incorporation of isotopes by mass accumulation. In order to focus on long-lived proteins and lipids, we limited our analysis to a single post-mitotic tissue, the indirect flight muscle, where the life span of molecules is not affected by cellular turnover. To determine whether the longevity of OXPHOS proteins is dependent on CL, we studied two *Drosophila* strains with enzyme deletions in the CL pathway.

## Results

### Parallel measurement of protein and lipid turnover in fruit flies

**Figure 1:**
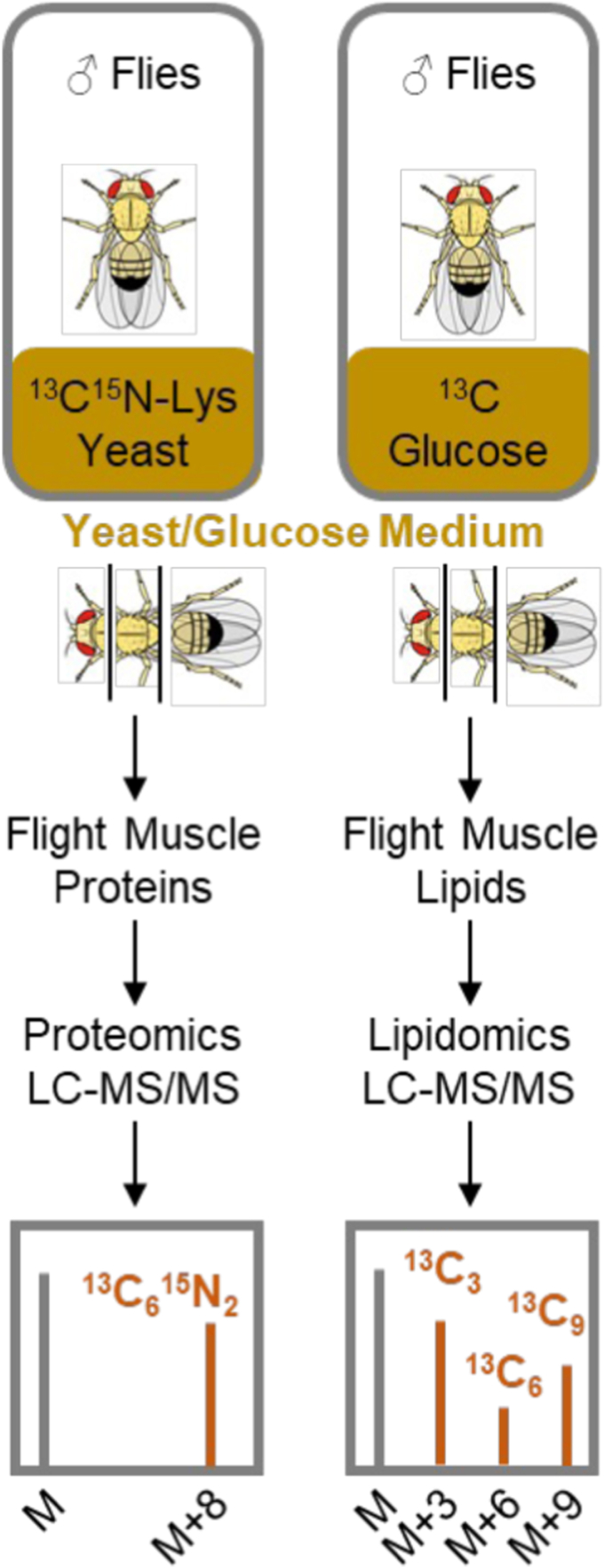
**Parallel measurement of protein and lipid turnover in *Drosophila* flight muscles.** Male flies were fed with yeast/glucose containing either heavy lysine or heavy glucose. Flight muscles were harvested at different time points (3–4 replicates per time point, 15 flies per replicate) and analyzed by LC-MS/MS on either a proteomics or lipidomics platform. Proteins and lipids were identified in MS2. Abundances of light and heavy isotopomers were measured in MS1.

We fed adult flies with one of two yeast/glucose media that contained identical chemical compositions but different isotopic labels. To measure protein turnover, the medium contained yeast labeled with ^13^C_6_^15^N_2_-lysine. To measure lipid turnover, the medium contained ^13^C_6_-glucose. Flight muscles were harvested by thorax excision after 2 and 4 days followed by the isolation of proteins or lipids. To generate peptides that contain a single lysine residue, we digested proteins by the protease LysC. Peptides and lipids were analyzed by liquid chromatography-tandem mass spectrometry (LC-MS/MS) on either a proteomics or a lipidomics platform. Molecular species were identified in MS2 and the abundances of light and heavy isotopomers were quantified in MS1 (Fig. 1).

The incorporation of lysine-derived isotopes into proteins created M + 8 isotopomers because each peptide contained only a single ^13^C_6_^15^N_2_-lysine residue. The incorporation of glucose-derived isotopes into lipids created multiple isotopomers because the metabolism of ^13^C-labeled glucose transferred ^13^C atoms into acyl, glycerol, and head group moieties. For lipids with a single glycerol moiety, we selected the M + 3 isotopomer for analysis because it contains ^13^C atoms only in the glycerol group and therefore reports specifically on the lipid backbone (14, 15). For phosphatidylglycerol (PG), we included the M + 3 and M + 6 isotopomers, and for CL, we included the M + 3, M + 6, and M + 9 isotopomers because those lipids contain 2 and 3 glycerol moieties, respectively (Fig. 1).

**Figure 2:**
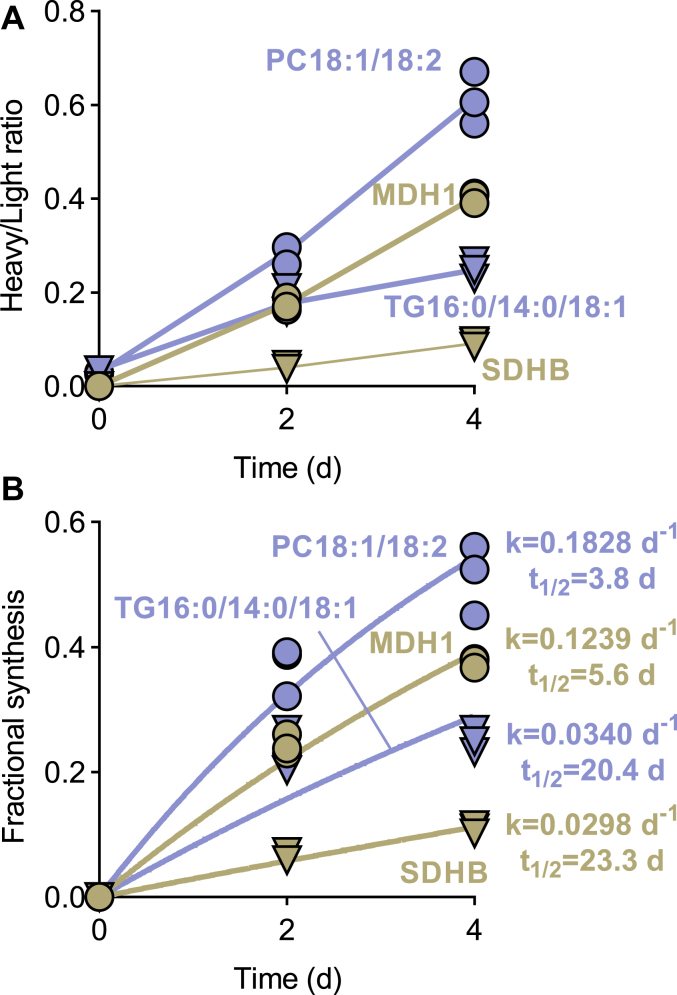
**Variable rates of incorporation of heavy isotopes into different proteins and lipids**. *A*, abundances of light and heavy isotopomers were measured in order to calculate heavy/light ratios. *B*, fractional syntheses were calculated from heavy/light intensity ratios. Turnover rate constants (k) were estimated by non-linear regression analysis. Half-life times (t_1/2_) were calculated from turnover rate constants. MDH1, mitochondrial malate dehydrogenase; PC18:1/18:2, oleoyl-linoleoyl-phosphatidylcholine; SDHB, subunit B of succinate dehydrogenase; TG16:0/14:0/18:1, palmitoyl-myristoyl-oleoyl-glycerol.

Since adult flies do not accumulate body mass over time and postmitotic flight muscles do not proliferate, the incorporation of heavy isotopes was solely caused by the intracellular turnover of proteins and lipids. We confirmed that the protein content of fly thoraces did not change during the incubation (8.3 ± 4.4 μg protein per thorax at day 0 and 9.6 ± 4.3 μg protein per thorax at day 4, N = 6, *p* = 0.614). To quantify turnover, we measured the ratio of the abundances of heavy/light (H/L) isotopomers at different time points. The progression of H/L ratios over time allowed us to discriminate between quickly metabolizing molecules, such as mitochondrial malate dehydrogenase or phosphatidylcholines, and slowly metabolizing molecules, such as mitochondrial succinate dehydrogenase or triglycerides (Fig. 2*A*).

To determine turnover rates from H/L ratios, it is necessary to account for the dilution of isotopes in the tissue due to the recycling of endogenous metabolites. Therefore, we used LC-MS/MS on a metabolomics platform to measure the isotope abundance in the metabolic precursors of proteins and lipids, which are lysine and glycerol-3-phosphate respectively, in flight muscle after feeding with the heavy media. With that information, we converted H/L ratios into fractional syntheses, that is, the portions of proteins and lipids replaced by newly synthesized molecules during the observation period. Fractional syntheses were plotted over time and analyzed by nonlinear regression in order to estimate rate constants of turnover and half-life times (Fig. 2*B*).

### Comparative half-lives of proteins and lipids in flight muscle

**Figure 3:**
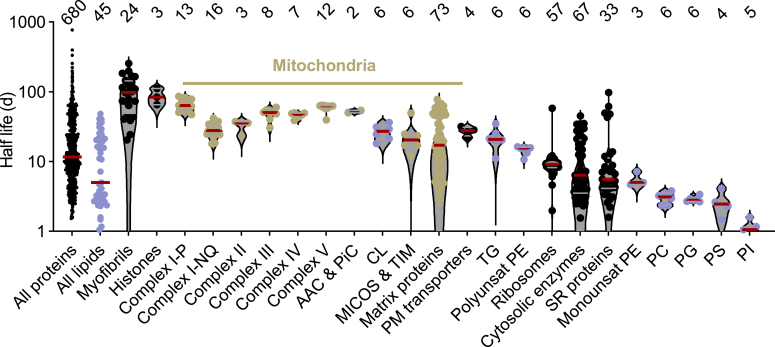
**Half-lives of groups of proteins and lipids**. Half-lives were calculated from rate constants. Columns show the half-lives of individual proteins and lipids and their median (*red line*). The sample size of each group is shown on top. AAC, ADP-ATP carrier; MICOS, mitochondrial contact site and cristae organizing system; PC, phosphatidylcholine; PE, phosphatidylethanolamine; PiC, phosphate carrier; PI, phosphatidylinositol; PM, plasma membrane; PS, phosphatidylserine; SR, sarcoplasmic reticulum; TG, triglyceride; TIM, translocase of the inner membrane.

We identified 680 proteins for which H/L ratios were reported in MaxQuant. In addition, we selected 45 lipids for which we used Xcalibur to extract H/L ratios from MS1 spectra. The half-lives of most proteins and lipids in flight muscle ranged from 1 to 100 days (Data S1). Among proteins, the mitochondrial complexes of OXPHOS had the longest half-life together with histones and myofibrils. Among lipids, CLs had the longest half-life. CLs and OXPHOS proteins, both residents of the mitochondrial inner membrane, lasted longer than most proteins in the plasma membrane, the cytosol, the sarcoplasmic reticulum, and ribosomes, as well as most lipids, including triglycerides (TG), phosphatidylcholines (PC), phosphatidylethanolamines (PE), phosphatidylserines (PS), phosphatidylinositols (PI), and PG. While lipid lifetimes were generally class-specific, we made the interesting observation that mono-unsaturated PE species turned over faster than polyunsaturated PE species (Fig. 3).

Within the OXPHOS system, the P module of complex I had the longest lifespan and the NQ module of complex I had the shortest. The long lifetimes of OXPHOS proteins (2, 5, 6, 16) and the discrepancy between the P and NQ-modules of complex I (7, 17) have already been described in mammals. Importantly, not all mitochondrial proteins were as stable as OXPHOS complexes. Many proteins in the mitochondrial matrix and two complexes of the inner boundary membrane, the protein translocase of the inner membrane (TIM) and the mitochondrial contact site and cristae organizing system (MICOS), had a much shorter lifespan. On the other hand, carriers for adenine nucleotides and phosphate, which reside in the crista, had long half-lives similar to OXPHOS proteins (Fig. 3). Together the data demonstrate a remarkable longevity of CLs, carriers, and respiratory proteins, suggesting that crista membrane domains harboring the OXPHOS system are relatively resistant to degradation.

### Effect of CL on the turnover of mitochondrial proteins and lipids

**Figure 4:**
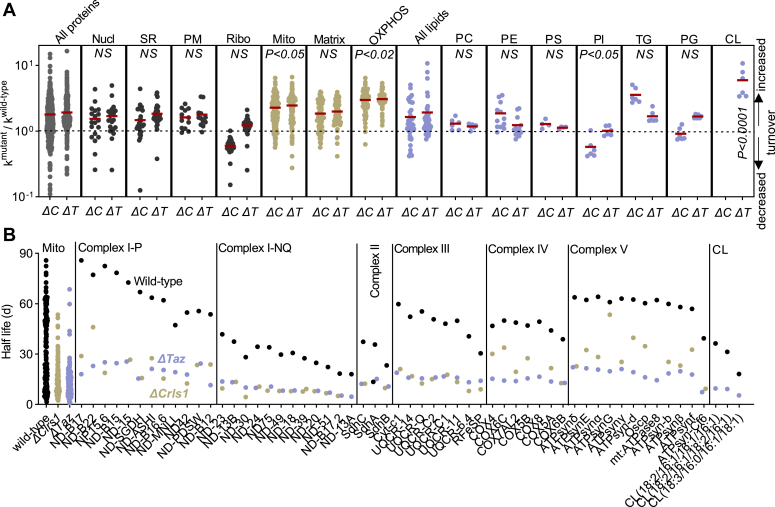
**Effect of CL deficiency on the turnover of proteins and lipids.***A*, data are mutant/wild-type ratios of the turnover rate constants of proteins and lipids. Red lines indicate means. Groups were compared by *t* test against the ratios of the entire proteome or lipidome, respectively. ΔC, *ΔCrls1*; ΔT, *ΔTaz*. *B*, data are half-lives of OXPHOS subunits and CL species in wild-type, *ΔCrls1*, and *ΔTaz*. The half-lives of all mitochondrial proteins are shown for comparison. In all groups (complexes I-V, CL), there was a significant difference between the wild-type and the mutants (*p* < 0.05; *t* test).

We measured protein and lipid turnover in two CL-deficient mutants, one with inactivated CL synthase (*ΔCrls1*, resulting in complete loss of CL) and one with inactivated tafazzin (*ΔTaz*, resulting in low concentration and abnormal composition of CL). Rate constants of individual proteins and lipids in the mutants were divided by the corresponding rate constants in the wild-type. In both mutants, CL deficiency decreased the turnover of some proteins (k^mutant^/k^wild-type^<1) but increased the turnover of others (k^mutant^/k^wild-type^>1). The average k^mutant^/k^wild-type^ ratio was 1.7 in *ΔCrls1* and 1.9 in *ΔTaz*, indicating a slight increase in the overall turnover of proteins as a result of CL deficiency. However, the turnover of OXPHOS proteins stood out in that it was about 3-fold higher in the mutants than in the wild-type. In contrast, proteins of the mitochondrial matrix and proteins of other cellular compartments did not show a large increase in turnover (Fig. 4*A*).

*ΔCrls1* and *ΔTaz* also caused a slight increase in the overall lipid turnover, resulting in k^mutant^/k^wild-type^ ratios of 1.6 and 1.9, respectively. Surprisingly, *ΔCrls1*, but not *ΔTaz*, decreased the turnover of PI. As reported previously by our laboratory (13, 18), *ΔTaz* strongly increased the turnover of non-remodeled CL, which confirmed that CL remodeling is essential for CL stability (Fig. 4*A*). Thus, our data indicate that CL deficiency increased the turnover of OXPHOS proteins and, in the case of *ΔTaz* where some CL remains, the turnover of CL itself.

Indeed, CL deficiency, caused either by *ΔCrls1* or *ΔTaz*, reduced the half-life of every OXPHOS subunit identified in the proteome. Proteins with the longest lifetime, such as the subunits of the P module of complex I and the subunits of complex V, were most affected. As a result of CL deficiency, all OXPHOS proteins and the remaining CL species assumed similarly short half-lives, largely eliminating any lifetime differences that were present in the wild-type (Fig. 4*B*). CL deficiency also shortened the lifetime of some matrix proteins, notably the long-lived enzymes of the Krebs cycle and proteins of the intermembrane space, such as cytochrome c and adenylate kinase (Data S1). Thus, the strong effect of CL on protein half-lives is not strictly a crista-specific phenomenon. Nevertheless, OXPHOS proteins were on average more affected by CL deficiency than matrix proteins (Fig. 4*B*). We are speculating that the instability of OXPHOS complexes had secondary effects on other proteins, mediated perhaps by mechanisms of mitochondrial quality control.

**Figure 5:**
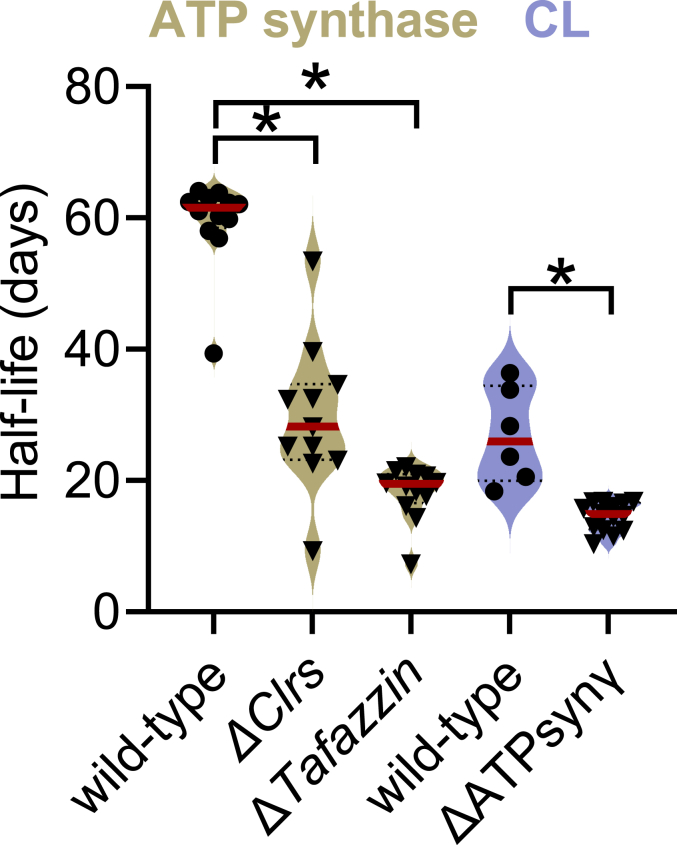
**Reciprocal effects of CL and ATP synthase on their half-lives in *Drosophila* flight muscle.** The graph shows half-lives of the subunits of ATP synthase (in wild-type, ΔCrls1, *ΔTaz*) and half-lives of CL species (in wild-type and a ATPsynγ-knockdown strain). Half-lives of ATP synthase were calculated from the present data, half-lives of CL species were calculated from data in reference 18. *Asterisks* indicate significant differences between groups (*p* < 0.0001, *t* test).

Importantly, the effect of CL on the lifetimes of OXPHOS proteins is reciprocal. We have previously shown that the broad downregulation of the expression of OXPHOS proteins increases the turnover of CL (18). Juxtaposing those data and the present ones reveals the bidirectionality of the effects on lifetimes between CL and OXPHOS proteins. As an example, we show here that ablation of CL shortened the half-life of the subunits of ATP synthase, while reduced expression of the ATP synthase shortened the half-life of CL (Fig. 5).

**Figure 6:**
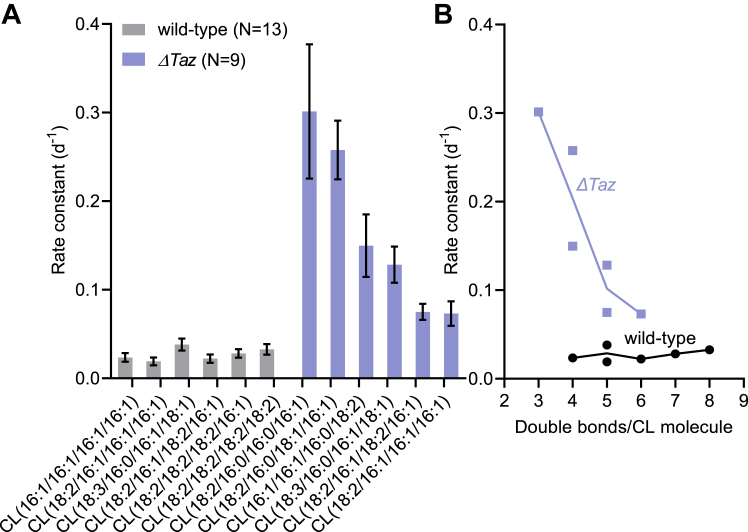
**Relation between unsaturation and turnover of CL species**. *A*, data are rate constants determined by non-linear regression with the 95% confidence interval. N is the number of data points used for regression analysis. *B*, data show the dependence of the rate constant on the number of double bonds per CL molecule. Each point represents a distinct molecular species.

Among the CL species that remained present in *ΔTaz*, saturated molecules had a higher turnover rate than unsaturated molecules (Fig. 6*A*). In fact, the turnover rate of CL species was inversely related to the number of double bonds per CL molecule in *ΔTaz* but not in the wild-type (Fig. 6*B*). These data demonstrate that unsaturated CLs remain partially protected even in the absence of remodeling, suggesting that CL unsaturation in and of itself is important for the lifetime of CL. We have provided evidence that unsaturated CL forms stronger associations with membrane proteins than saturated CL (13), which could explain the difference between unsaturated and saturated CL species in *ΔTaz*.

## Discussion

Several articles have stressed that OXPHOS subunits belong to the most stable proteins of the cell (2, 5, 6, 7, 8, 16) but the mechanism that confers stability to OXPHOS proteins in the dynamic environment of mitochondria, has not been established. Given the wide range of lifetimes within the mitochondrial proteome and even within individual complexes, selective quality control pathways must be at work in order to remove subsets of proteins while preserving the presence of others (19). One example is the selective degradation of the N module of complex I by the matrix protease ClpXP (17). This mechanism allows the continuous regeneration of one of the most labile sub-complexes while avoiding the laborious task of rebuilding the entire complex. However, it is not clear what confers resistance against ClpXP or other proteases to the membrane-embedded P module of complex I or indeed to other long-lived subunits of the OXPHOS system.

Our data indicate that CL is involved in the longevity of OXPHOS proteins. Either the loss of CL remodeling (*ΔTaz*) or the lack of CL (*ΔCrls1*), exposed OXPHOS proteins to increased turnover. The effect was stronger on the membrane-embedded P module of complex I than on the peripheral N module of complex I, which is consistent with the intramembranous localization of CL and suggests that CL controls protein lifetimes through changes in the membrane environment. Multiple effects of CL have been reported on mitochondrial membranes. For instance, CL deficiency reduces membrane potential (20) and the propensity to form respiratory supercomplexes (21, 22). CL deficiency also reduces the density of OXPHOS proteins in the inner mitochondrial membrane (23), which not only decreases the respiratory capacity of mitochondria but also changes the physical properties of the inner membrane because protein crowding shifts the phase state of the membrane from fluid to quasi-crystalline.

We argue that the presence of densely packed cristae could be sufficient to explain longevity. Crowding imparts strong lipid-protein and protein-protein interactions while it limits diffusion and molecular motion. Strong interactions and slow diffusion are likely to restrict the exposure to proteases and lipases, which would prolong the lifetimes of proteins and lipids. Our data strongly support a mechanism based on lipid-protein interactions because we found co-dependence of the longevities of OXPHOS proteins and CL. Specifically, deficiency of CL shortened the half-life of OXPHOS proteins while deficiency of OXPHOS proteins shortened the half-life of CL. The idea of densely packed OXPHOS domains is also supported by published data showing restricted diffusion of OXPHOS complexes, that is, individual cristae remain intact after mitochondrial fusion until they are gradually blended by slow diffusion (24).

To understand the effect of CL, one has to bear in mind its strong but non-specific affinity to proteins, which seems to result from the unique combination of a small inflexible head group and a large flexible hydrophobic moiety (25). The rigid head group diminishes the capacity for shielding the phosphate groups, which exposes them to ionic interactions with proteins, while the flexible hydrophobic moiety confers adaptability to different types of protein surfaces. The size of the hydrophobic moiety is important because monolyso-CL, a variant of CL with only 3 acyl chains, is less effective in interacting with respiratory complexes (13, 26). Therefore, the accumulation of monolyso-CL in tafazzin-deficient mitochondria, cannot compensate for the loss of CL.

Our data carry particular significance for insect flight muscles, a specialized organ that performs one of the most demanding locomotor activities in nature (27, 28). To sustain wing-beat frequencies of more than 100 per second, flight muscles require a huge oxidative capacity, for which they need to concentrate more OXPHOS complexes within a given volume than most other tissues (29, 30). The high abundance of cytochromes within flight muscles and the correlation between cytochrome content and wing-beat frequency have been recognized for many years (31), attesting to the critical importance of the OXPHOS system and its functional involvement with flight. Conferring longevity to the OXPHOS system avoids the need to continuously regenerate elaborate machinery with all the associated costs. However, the combination of a high enzymatic turnover number (high enzyme activity) with a low turnover of the enzyme (long half-life) may lead to an excessive number of catalytic cycles that each enzyme molecule has to perform during its lifetime. Theoretically, this may become a liability if proteins suffer wear-and-tear damages but are not being replaced.

In summary, we have measured side-by-side the half-lives of proteins and lipids in a single post-mitotic tissue, which enabled direct comparisons between the lifetimes of protein and lipid species *in vivo*, including those that are co-localized in the inner membrane of mitochondria. We confirmed the longevity of CL and OXPHOS complexes and demonstrated that one is dependent on the other. By showing that CL is essential for the longevity of OXPHOS proteins we discovered perhaps one of the most important functions of CL in mitochondria.

## Experimental procedures

### Fly strains

*Drosophila* strains were maintained on standard fly food containing yeast, cornmeal, and molasses in 3-inch culture vials at 24 °C. The strain w^1118^; PBac{PB} CG4774^c01874^/TM6B, Tb1, with a transposon insertion in the coding region of the last exon of the CL synthase gene (*ΔCrls1*), was obtained from the Bloomington *Drosophila* Stock Center (No. 10741). The tafazzin mutant (*ΔTaz*) was created in our laboratory (32). To avoid confounding effects, we re-derived all strains in identical genetic backgrounds (23).

### Fly media containing stable isotopes

The fly food used to label *D. melanogaster* was adopted from a previous publication (33). The medium consisted of 150 mg glucose and 30 mg yeast paste, which were mixed in a small amount of water and placed on a 0.25 inch × 0.25 inch square of filter paper. For the measurement of protein turnover, ^13^C_6_^15^N_2_-lysine-labeled yeast was mixed with unlabeled glucose. For the measurement of lipid turnover, unlabeled yeast was mixed with ^13^C_6_-glucose. Stable isotopes were obtained from Cambridge Isotope Lab. Inc. (Andover, MA 01810 USA). To prepare labeled or unlabeled yeast, the lysine auxotrophic yeast strain SUB62/DF5 (MATalpha lys2-801 leu2-3/112 ura3-52 his3-delta200 trp1-1), obtained from the American Type Culture Collection (ATCC #200912), was grown on a medium containing 1.7 g/L yeast nitrogen base (without amino acids, without ammonium sulfate), 20 g/L D -glucose, 5 g/L ammonium sulfate, 200 mg/L adenine hemisulfate, 20 mg/L uracil, 100 mg/L tyrosine, 10 mg/L histidine, 60 mg/L leucine, 10 mg/L methionine, 60 mg/L phenylalanine, 40 mg/L tryptophan, 100 mg/L arginine, and 30 mg/L ^13^C_6_^15^N_2_-lysine or unlabeled lysine, respectively. LC-MS/MS analysis demonstrated that >99% of proteins were labeled when yeast cells were grown in the presence of heavy lysine.

### Incorporation of stable isotopes

In order to measure the turnover of proteins and lipids, flies were kept on media that contained either heavy lysine or heavy glucose. To reduce variability, only male flies were studied. Flies were kept at 24 °C in a moisturized chamber containing the glucose/yeast media specified above. Batches of 15 animals were collected per measurement. Flies were harvested after 0, 2, and 4 days. Thoraces were dissected under the microscope and stored at −80 °C until further use. Thoraces were homogenized in water and processed for protein or lipid analysis.

### Protein analysis

Proteins were purified and concentrated by a brief SDS-PAGE run designed to focus all proteins into a single band of about 1 cm × 0.5 cm. Gels were washed 3 times in double-distilled water for 15 min each. Proteins were visualized and fixed by staining with EZ-Run Protein staining solution (ThermoFisher Scientific). The stained protein gel regions were excised and de-stained 3 times in 50% methanol for 15 min each time. Then the gel regions were treated with 50 mM NH_4_HCO_3_ in 30% acetonitrile until completely de-stained. Finally, each gel sample was dehydrated with 100% acetonitrile. Dried gel samples were digested overnight with mass spectrometry grade Endoproteinase Lys-C (Mass Spec Grade, Promega) at 25 ng/μl in 50 mM NH_4_HCO_3_ digest buffer. After acidification with 10% formic acid, peptides were extracted with 5% formic acid/50% Acetonitrile (v/v) and concentrated to a small droplet using vacuum centrifugation. Desalting of peptides was done using hand-packed SPE Empore C18 Extraction Disks as described (34). Desalted peptides were again concentrated and reconstituted in 10 μl 0.1% formic acid in water. Aliquots of the peptides were analyzed by nano-liquid chromatography followed by tandem mass spectrometry (nano-LC-MS/MS) using an Easy nLC 1000 equipped with a self-packed 75 μm x 20 cm reverse phase column (ReproSil-Pur C18, 3 μm, Dr Maisch GmbH) coupled online to a QExactive HF Orbitrap mass spectrometer *via* a Nanospray Flex source (all instruments from Thermo Fisher Scientific). Analytical column temperature was maintained at 50 °C by a column oven (Sonation GmBH). Peptides were eluted with a 3 to 40% acetonitrile gradient over 110 min at a flow rate of 250 nl/min. The mass spectrometer was operated in data-dependent analysis mode with survey scans acquired at a resolution of 70,000 (at m/z 200) over a scan range of 300 to 1750 m/z. Up to 10 of the most abundant precursors from the survey scan were selected with an isolation window of 1.6 Th for fragmentation by higher-energy collisional dissociation with a normalized collision energy of 27. The maximum injection times for the survey and MS/MS scans were 60 ms and the ion target value for both scan modes was set to 3e6. Data were analyzed by MaxQuant software (version 1.5.5.1). We used the Andromeda search engine (35, 36) to search the Uniprot *Drosophila* protein sequence database (downloaded on 01/09/2015; 3214 entries).

### Lipid analysis

Lipids were extracted into chloroform/methanol as described (37). To this end, samples were homogenized in 0.2 ml water, suspended in methanol/chloroform (2:1), and incubated at 37 °C for 30 min to denature proteins. Chloroform and water were added, the samples were vortexed, and phase separation was achieved by centrifugation. The lower phase was collected, dried under nitrogen, and re-dissolved in 0.2 ml chloroform/methanol (1:1). Lipids were analyzed by LC-ESI-MS/MS on a QExactive HF-X instrument coupled directly to a Vanquish UHPLC (Thermo Fisher Scientific). An aliquot of 5 μl was injected into a Restek Ultra C18 reversed-phase column (Restek Corporation; 100 × 2.1 mm; particle size 3 μm) that was kept at a temperature of 50 °C. Chromatography was performed with solvents A and B at a flow rate of 0.15 ml/min. Solvent A contained 600 ml acetonitrile, 399 ml water, 1 ml formic acid, and 0.631 g ammonium formate. Solvent B contained 900 ml 2-propanol, 99 ml acetonitrile, 1 ml formic acid, and 0.631 g ammonium formate. The chromatographic run time was 40 min, changing the proportion of solvent B in a non-linear gradient from 30 to 35% (0–2 min), from 35 to 67% (2–5 min), from 67 to 83% (5–8 min), from 83 to 91% (8–11 min), from 91 to 95% (11–14 min), from 95 to 97% (14–17 min), from 97 to 98% (17–20 min), from 98 to 100% (20–25 min), and from 100 to 30% (25–26 min). For the remainder of the run time the proportion of solvent B stayed at 30% (26–40 min). To analyze triglycerides, the mass spectrometer was operated in positive ion mode. For all other lipids, it was operated in negative ion mode. The spray voltage was set to 4 kV and the capillary temperature was set to 350 °C. MS1 scans were acquired in profile mode at a resolution of 120,000, an AGC target of 1e6, a maximal injection time of 65 ms, and a scan range of 200 to 2000 m/z. MS2 scans were acquired in profile mode at a resolution of 30,000, an AGC target of 3e6, a maximal injection time of 75 ms, a loop count of 7, and an isolation window of 1.7 m/z. The normalized collision energy was set to 30 and the dynamic exclusion time to 31 s. For lipid identification, data were analyzed by the software LipidSearch 5.0 (Thermo Fisher Scientific). The general database of LipidSearch 5.0 was searched with a precursor tolerance of 4 ppm, a product tolerance of 10 ppm, and an intensity threshold of 1.0%.

### Metabolite analysis

To analyze water-soluble metabolites, thorax samples were homogenized in 100 μl methanol/water (8 + 2) and kept at −80 °C overnight. Homogenates were spun for 5 min at 15,000 g in Eppendorf tubes. The supernatants were analyzed on a QExactive HF-X mass spectrometer directly coupled to a Vanquish UHPLC system (ThermoFisher Scientific). A 5-μL aliquot of the supernatant was injected onto a 5 μm C18 Acclaim 120 column (4.6 × 100 mm, ThermoScientific). Metabolites were eluted with a 5 to 50% methanol gradient in water containing 0.1% formic acid and 0.2 g/l ammonium acetate over 50 min at a flow rate of 1 ml/min. The mass spectrometer was operated in positive ion mode and data-dependent mode with survey scans acquired at a resolution of 120,000 over a scan range of 100 to 1000 m/z. Up to five of the most abundant precursors from the survey scan were selected with an isolation window of 1.7Th and fragmented by higher-energy collisional dissociation with a normalized collision energy of 30. The maximum ion injection time for the survey and MS/MS scans was 75 ms and the ion target value for MS and MS/MS scans were set at 3e6 and 1e5, respectively. The labeling of water-soluble metabolites was determined in MS1 scans acquired from 0.97 to 1.07 min. To determine the relative abundance of heavy lysine, we measured the intensities of the monoisotopic isotopomer and the ^13^C_6_^15^N_2_ isotopomer. To determine the relative abundance of heavy glycerol-3-phosphate, we measured the intensities of the monoisotopic isotopomer and the ^13^C_3_ isotopomer.

### Calculation of turnover rate constants and half-life times

The principles of turnover analysis were previously described for proteins (38) and lipids (15). Protein mass spectra were processed by the software MaxQuant (version 1.5.5.1) to yield heavy/light abundance ratios (*r*) for each identified protein. The ratios were converted into relative isotopomer abundances (*r/1+r*) and divided by the relative abundance of heavy lysine in the free lysine pool [*p* = labeled lysine/(labeled lysine + unlabeled lysine)] in order to yield the fractional synthesis (*q*), *i.e.* the proportion of newly synthesized protein in the total amount of protein present at time *t*:

$$q_{t}=\frac{r_{t}}{p_{t}\left(1+r_{t}\right)}$$

The relative isotopomer abundance was divided by *p* in order to correct for the presence of unlabeled lysine in the free lysine pool, which dilutes the abundance of heavy isotopes in newly synthesized peptides. Turnover rate constants (k) were estimated from fractional syntheses measured at different time points (*t*) by non-linear regression to the equation:

$$q_{t}=1-e^{-kt}$$

To use this equation, we assumed that the total concentration of a molecule, *i.e.* the sum of labeled and unlabeled species, did not change during the experiment. Half-life times were calculated as:

$$t_{1/2}=\frac{\ln\;2}{k}$$

Lipid mass spectra were processed in the Qual Browser of Xcalibur (version 4.1.50). Lipid species with favorable signal-to-noise ratios were manually selected for analysis. In most lipids, we measured the abundance of the light isotopomer (monoisotopic peak) and the abundance of the heavy isotopomer (^13^C_3_ peak) and applied the same data processing algorithm as for proteins. In the lipid work-flow, *p* is the relative abundance of ^13^C_3_-glycerol-3-phosphate in total glycerol-3-phosphate [*p* = labeled G-3-P/(labeled G-3-P + unlabeled G-3-P)]. In case of PG and CL, several isotopomers were measured and the data were processed as described (15).

## Data availability

The raw mass spectrometry data of the proteomics analysis are available at MassIVE (UCSD) and can be accessed at https://massive.ucsd.edu/ProteoSAFe/static/massive.jsp the dataset identifier MSV000091946.

## Supporting information

This article contains supporting information.

## Conflict of interest

The authors declare that they have no conflicts of interest with the contents of this article.

## Supporting information

Data Set 1
